# Twenty Years of Experience with the Preoperative Diagnosis of Medullary Cancer in a Moderately Iodine-Deficient Region

**DOI:** 10.1155/2013/571606

**Published:** 2013-03-06

**Authors:** Tamas Solymosi, Gyula Lukacs Toth, Dezso Nagy, Istvan Gal

**Affiliations:** ^1^Thyroid Outpatient Department, Bugat Hospital, 6 Fenyves Street, Matrafured, Gyongyos 3232, Hungary; ^2^Department of Pathology, Bugat Hospital, Dozsa Gyorgy Street, Gyongyos 3200, Hungary; ^3^Department of Nuclear Medicine, Honved Hospital, 44 Robert Karoly Avenue, Budapest 1134, Hungary; ^4^Department of Surgery, Telki Hospital, Telki 2089, Hungary

## Abstract

*Background*. There is a current debate in the medical literature about plasma calcitonin screening in patients with nodular goiter (NG). We decided on analyzing our 20-year experience with patients in an iodine-deficient region (ID). *Patients and Methods*. 22,857 consecutive patients with NG underwent ultrasonography and aspiration cytology (FNAC). If FNAC raised suspicion of medullary cancer (MTC), the serum calcitonin was measured. *Results*. 4,601 patients underwent surgery; there were 23 patients among them who had MTC (0.1% prevalence). Significantly more MTC cases were diagnosed cytologically in the second decade than in the first: 11/12 and 6/11, respectively. The frozen section was of help in 2 cases out of 3. Two patients suffered from a 3-year delay in proper therapy, and reoperation was necessary in 1 case. FNAC raised the suspicion of MTC in 20 cases that were later histologically verified and did not present MTC. The diagnostic accuracy of FNAC in diagnosing MTC was 99.2%. Two false-positive serum calcitonin tests (one of them in a hemodialyzed patient) and one false-negative serum calcitonin test occurred in 40 cases. *Conclusion*. Regarding the low prevalence of MTC in ID regions, calcitonin screening of all NG patients does not only appear superfluously but may have more disadvantages than advantages.

## 1. Introduction

Medullary thyroid carcinoma (MTC) is accounted for 3.5–10% of all thyroid malignancies [1–3]; the lesions are derived from the parafollicular C-cells which produce calcitonin [4]. As for all thyroid nodules, the preoperative diagnosis is primarily based on ultrasonography (US) and fine needle aspiration cytology (FNAC); however, there are conflicting data about the usefulness of FNAC in the diagnosis of MTC [5, 6]. The reported sensitivity of FNAC for the diagnosis of MTC is less than that for the diagnosis of papillary cancer, but the broadness of the range—from 40 to 88%—may reflect differences in skills of the cytopathologists [5–8]. 

On the one hand, the serum calcitonin level is considered to be the most sensitive and specific marker of MTC; on the other hand, it must be taken into consideration that not all MTCs produce calcitonin [9], and hypercalcitoninemia is known to be associated with chronic thyroiditis and C-cell hyperplasia besides MTC [8]. 

There is an ongoing debate in the medical literature about benefits from routine measurement of plasma calcitonin levels in patients who have thyroid nodules [10]. The new AACE/AME/ETA guidelines regarding the diagnosis and management of thyroid nodules state that “single, non-stimulated calcitonin measurement can be used in the initial workup of thyroid nodules and it is recommended before thyroid nodule surgery” [11]. Most of the publications serving as the basis of these guidelines are related to iodine-sufficient regions, and this factor led us to analyze our 20 years of experience in a moderately iodine-deficient region. 

## 2. Patients and Methods

During the 20-year period of 1991–2011, all patients, who were present at our thyroid outpatient department for the first time, underwent ultrasonography and TSH determination. 22,857 patients with nontoxic thyroid nodular goiters underwent FNAC. Patients with a solitary nodule larger than 1 cm in the maximal diameter underwent US-guided FNAC. The largest nodule was aspirated in the presence of multinodular goiters; if that was not hypoechogenic, the largest hypoechogenic nodule was aspirated too. Hypoechogenic nodules containing small hyperechogenic granules—and/or those with ill-defined borders larger than 5 mm—were also aspirated.

We used the following categories in cytological analysis: not diagnostic, benign, suspicious (including follicular tumors), and malignant. We also provided the subtype of the possible malignant disease with the last two categories.

When FNAC raised the suspicion of MTC or a malignancy including MTC, the serum calcitonin level was determined.

The clinical appearance was analyzed in the cases of all our patients with nodular goiter. The classification was a clinical suspicion of malignancy in the presence of recurrent palsy with causes determined only inside the thyroid, a hard nodule with an uneven surface except for cases which were caused by calcification, or a rapidly growing firm or hard thyroid mass except for cysts.

Between 1991 and 1997, the serum calcitonin level was determined by radioimmunoassay (RIA); however, after this time period, we used an immunoradiometric (IRMA) calcitonin assay (normal range 0.8–9.9 pg/mL).

4,601 patients underwent surgery which was indicated by a positive cytological report, a clinical suspicion of malignancy, compression signs, or because of the wish of the patient. Histologically diagnosed MTC cases were confirmed by a positive immunohistochemical reaction with calcitonin and chromogranin A: immunostaining of tumor cells with anticalcitonin and with chromogranin A was positive, while the results with antithyroglobulin test were negative in all 23 patients with the presence of MTC. All patients who were not operated on were participating in regular followup examinations with TSH, US every year, and repeated FNAC in case the nodule increased in volume by more than a third. 86.1% of these patients were present for at least one examination within 5 years. 

## 3. Results

### 3.1. Distribution by Sex

The proportion of men in the MTC group (30.4%) is proved to be significantly higher than in the non-MTC group (12.9%) or in the benign nodular goiter group (9.2%), *P* < 0.05 (CF = 4.43) and *P* < 0.01 (CF = 9.75), respectively (Table 1). The average age of MTC patients was 53.1 ± 12.6 years.

### 3.2. Clinical Presentation of MTC

The clinical presentation was more often indicative in MTC patients (43%) than in non-MTC patients (26%, *P* = 0.11) or in those with benign nodular goiter (4.1%), *P* < 0.001 (CF = 75.9). The clinical presentation raised the suspicion of malignancy in 10 of the 23 MTC cases including 1 patient with a benign FNAC result. Among 10 patients with a clinical suspicion of malignancy, 3 had palsy of the recurrent nerve with causes determined exclusively within the thyroid. Seven patients had hard nodules with an uneven surface, and 9 indicated a rapidly growing firm or hard thyroid mass among 19 patients with palpable nodules.

### 3.3. US Findings in MTC

**Figure 1:**
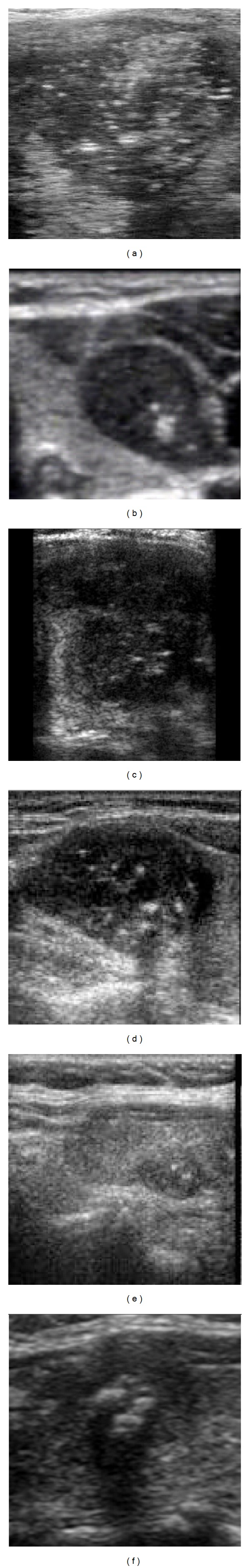
Ultrasonography of medullary cancer. (a)–(c) medullary cancer, (d)-(e) papillary cancer, (f) oxyphilic adenoma. Compare the large irregular patchy hyperechogenic foci in images (a)–(d) with the few brightSmall spots of microcalcifications < than 1 mm in maximal diameter in image (e). In contrast to macrocalcifications (image (d) and (f)), they do not indicate dorsal acoustic shadow according to the entire extent of the hyperechogenic focus.

All 23 MTC cases involved hypoechogenic nodules. The most specific sign of MTC with a sensitivity of 47% and an odd ratio of 31.5 (95% confidence interval 15–66) was the presence of multiple amorphous hyperechogenic foci within the nodule (see Figure 1). These foci were larger than 1 mm in diameter in contrast to microcalcifications. They displayed an irregular patchy appearance and did not indicate a dorsal acoustic shadow according to the entire extent of the hyperechogenic focus as opposed to coarse calcifications. This feature was observed in 11 out of the 23 histopathologically diagnosed MTC and in 67 out of the 3,988 other patients who underwent surgery. There were two other findings which were discovered and also statistically more often occurring in MTC than in benign lesions: the ratio of the horizontal to the anteroposterior diameter was greater in MTC and a remarkably higher proportion of the nodules had irregular (spiculated) or blurred borders. We do not report details of these properties as of the lack of their practical significance (positive predictive value < than 3%) in the differential diagnostics.

#### 3.3.1. Cytological Diagnosis of MTC

Results of cytological examinations were positive (either malignant or suspicious of malignancy) in all 23 MTC cases except for 2 cases that occurred in the first 10-year period (Table 2). It is important to note that FNAC raised the suspicion of MTC in only 6 cases out of 11 in the first 10 years; however, the FNAC result was correct in 11 out of the 12 cases in the second 10-year period. The difference was statistically significant (*P* = 0.04, chi-square test). See next section for further details. 

There were 12 cases in the first decade and 9 cases in the second decade when the FNAC raised the suspicion of the possibility of MTC, although the final histopathological results did not prove the presence of MTC. The calcitonin level was <10 pg/mL in all cases except for two patients. In 12 of 21 cases, Hürthle-cell tumor caused differential diagnostic problems which were later verified histopathologically. In 4 other cases, the atypical lymphoid population caused issues. (The final histopathological findings were the following: 1 MALT lymphoma, 2 Hashimoto's thyroiditis, and 1 papillary cancer coexisting with Hashimoto's thyroiditis.) The origins of the atypical cells on the smear were presented by a different diagnostic problem in the remaining 5 cases. (The final histopathology findings were the following: 1 anaplastic cancer, 1 Hürthle-cell cancer, 1 metastatic cancer, 1 Hürthle-cell adenoma, and 1 hyalinizing trabecular adenoma.)

The sensitivity of FNAC for diagnosing MTC was 74% (17/23), and the specificity was 99.6% (4,560/4,578), while the diagnostic accuracy was 99.5% (4,577/4,601).

### 3.4. Results of Calcitonin Determination

The calcitonin level was in the range 8–2,552 pg/mL with a median level of 277 in 19 preoperatively diagnosed MTC cases (including 2 patients whose disease was diagnosed only on the second occasion). In 1 patient with MTC with a distant metastasis, the calcitonin level measured with the RIA method was falsely negative (8 pg/mL). Three other patients demonstrated a calcitonin level <100 pg/mL (38, 77 and 91 pg/mL, resp.). 

The calcitonin level was <10 pg/mL in 19 out of 21 cases where FNAC raised the possibility of MTC; however, the final histopathology ruled that option out. 

One false-positive calcitonin result (118 pg/mL with the RIA method) occurred in the case of a Hürthle-cell cancer and another case was noted in the case of an adenoid cystic cancer of a small salivary gland metastasizing to the thyroid (706 pg/mL with the IRMA method). The latter patient had chronic renal failure and was also hemodialyzed. Total thyroidectomy was performed in both cases. Detailed histopathological analysis did not detect either MTC or C-cell hyperplasia in these 2 cases. The serum calcitonin level became normal after thyroidectomy in both cases. It means that the extrathyroidal origin of the elevated calcitonin level can be precluded from the perspective.

#### 3.4.1. Patients in Whom the First Evaluation Missed the Diagnosis of the Presence of MTC

We encountered 5 patients during the first 10-year period, while the second period revealed only 1 further patient with the same problem.

*Patient 1.* FNAC indicated colloid goiter. (A review of the smear revealed an error in gaining material.) The follow-up examination showed that the nodule had increased in size, and repeated FNAC pointed out the presence of MTC. 

*Patient 2.* FNAC resulted in a diagnosis of Hürthle-cell tumor. (The review of the smear indicated an error in interpretation.) The patient refused surgery. The nodule had increased in size after three years, and MTC was diagnosed cytologically. 

*Patient 3.* FNAC raised suspicion of malignancy. It might possibly be Hürthle-cell tumor or insular cancer as well. (The review of the smear suggested an error in the interpretation, and the possibility of MTC had to be taken into consideration.) The frozen section examination led us to the diagnosis of malignancy. The calcitonin level was high of 177 pg/mL after total thyroidectomy and histopathological diagnosis of MTC. Enlarged lymph nodes were detected in the medial compartment of the neck on the CT scan, and the patient was operated again. 

*Patient 4.* FNAC resulted in a diagnosis of Hashimoto's thyroiditis although the clinical presentation raised suspicion. (The review of the smear indicated an error of gaining material.) The patient underwent surgery. The frozen section diagnosis showed the presence of MTC. The surgical procedure was extended to the medial compartment of the neck. The final histopathology indicated MTC and Hashimoto's thyroiditis. 

*Patient 5.* FNAC indicated Hürthle-cell tumor. The final histopathology was Hürthle-cell carcinoma (25 mm in maximal diameter) and a 3 mm incidental focus of MTC. The postoperative serum calcitonin level was normal. The patient feels well and totally recovered 15 years after the surgical procedure. 

*Patient 6.* FNAC led us to the suspicion of malignancy. (The review of the smear suggested an error in the interpretation, and the possibility of MTC arose.) The frozen section diagnosis indicated carcinoma with a high probability of MTC. The surgical procedure was extended to the medial compartment of the neck. 

## 4. Discussion

The prevalence of MTC among our nodular goiter patients was 0.1% which is identical with published results without calcitonin screening [12] and less than the reported amount of 0.18–0.85% based on calcitonin screening [2, 6, 8, 12–19]. 0.49% of the patients who underwent surgery showed MTC on histopathology which is also lower than most published data, varying between 0.58 and 1.12% [5, 20, 21]. 

Differences in prevalence may be explained by the well-known goitrogenous effect of iodine-deficiency, which increases the number of benign nodules but not the malignant ones [22]. The proportion of MTC among thyroid cancers is not influenced by the iodine intake. This proportion was 5% in our series which is still within the previously published range of 1.4–15.7% [5, 6, 18, 20, 23–28]. 

The question arises about the advantages and disadvantages that would have emerged if we had used calcitonin screening in all our cases or in all of the surgically treated nodular goiter patients.

Two out of the 23 patients lost 3 years before the proper treatment was started. Nevertheless, the delay was not our responsibility in one of these cases as we advised surgery right after the false diagnosis of a Hürthle-cell tumor. Besides, there was another patient for whom reoperation could have been avoided. The question arises again: how many additional MTC cases would have been found if we had used calcitonin screening? 

If we extrapolate the 1 missed MTC that was larger than 5 mm in maximal diameter among the 4,601 operated patients compared to the 18,256 patients who were not treated surgically, this would have meant 4 more MTC cases. If we consider situations when we performed repeated FNAC in the presence of growing nodules and also the fact that no MTC cases occurred when the first and the second FNACs were negative, we can conclude that the appropriate number of clinically relevant missed MTCs were surely less than 4 cases. 

The situation differs concerning micro-MTCs. It seems to be obvious that we would have found more micro-MTC cases if we had performed calcitonin screening. Nevertheless, the clinical significance of occult MTC is not clear, therefore, we agree with other specialists that the occurrence of untreated occult MTC without morbidity or mortality should be considered in cost-effective models of routine serum calcitonin screening [29, 30]. 

Our findings also proved that the calcitonin test became safer with time. Two false results (1 false-positive and the only 1 false-negative calcitonin test) occurred with the RIA, while 1 false-positive emerged with the IRMA method. The latter occurred in a hemodialyzed patient with renal failure. Elevated calcitonin levels have been described in both cases in up to 25% of the former ones [31]. Although the new IRMA method (as in our practice) is clearly superior to the previously used RIA technique as the sensitivity and specificity of calcitonin screening with that method had been reported to be 75% and 98%, respectively [18, 32–39], the use of calcitonin screening to examine all nodular or all surgically treated patients—in case we assume an only 1% false positivity rate—would have meant 182 unnecessary operations or 46 operations with unnecessary radicalism.

If we consider the advantages and the disadvantages, it seems possible that in our cases the disadvantages of calcitonin screenings may have exceeded the advantages of the method. It has been clearly demonstrated that the cost-efficiency of calcitonin screening is highly dependent on the prevalence of MTC and the specificity of FNAC. In a decision model for a hypothetical group of patients with a 0.78% prevalence of MTC, calcitonin screening seemed to be cost-effective [32]. In the presence of iodine deficiency with a lower prevalence of MTC, the cost-efficiency of calcitonin screening would be lower. 

The aggressive clinical presentation was a typical but not specific sign of MTC; however, it led to the correct therapy in spite of the false-negative FNAC in the case of 1 patient.

We have found one specific US sign for MTC which may be of practical relevance: the presence of irregular patchy hyperechogenic areas within hypoechogenic nodules—which were observed in 48% of our MTC cases—increased the possibility of MTC diagnosis more than 30 times. These foci are larger than 1 mm, and they are not so bright in contrast to microcalcifications. They also have an even patchy appearance, and the dorsal acoustic shadow is absent or narrower than the extent of the hyperechogenic focus. We agree with Gorman et al. that these foci are not simply coarse calcifications but correspond to deposits of calcium surrounded by amyloid [40].

FNAC raised the possibility of a malignant disease in 21 cases out of the 23 (91% sensitivity). The average sensitivity for diagnosing MTC in our practice was 74%, though there was significant difference between our skills in the two time periods. The sensitivity was only 55% in the first 10-year period; however, it was 92% in the second 10-year period. As regards the opposite situation, we raised the possibility of MTC in 21 cases where the final histology was not MTC. The main problem in the cytological differential diagnostic was caused by Hürthle-cell tumors [41]. On the other hand, if we analyze our cases with proven Hürthle-cell tumors, MTC caused a differential diagnostic problem in only 1.5% of these cases. 

Our results emphasize the role of the frozen sections in those patients where the FNAC resulted in the suspicion of malignancy which was not otherwise specified [16]. The use of frozen sections in such cases corrected the insufficiency of the preoperative diagnosis in 2 among 3 patients. There were no MTC cases among the 361 patients whose FNAC was not repeatedly diagnostic, so this situation is not an indication for calcitonin testing. 

All our patients had sporadic form of MTC except for one person. The low proportion of familiar form may be explained by the fact that our MTC patients after surgery are treated in university centers, and the evaluation of relatives of familiar MTC patients is also done in these centers. 

*To Summarize.* The following protocol seems to be adequate in an iodine-deficient region with only a 0.1% prevalence of MTC: the performance of US-guided FNAC in all cases of hypoechogenic nodules, the testing of serum calcitonin only in cases where FNAC raised the possibility of MTC besides patients at risk of the familiar form of MTC, and also the performance of frozen sections in those cases where FNAC leads to the suspicion of malignancy which was not otherwise specified. It is even more important that thyroid FNAC should be carried out only by a cytologist with special interest and skills in this field for the evaluation of MTC. In evaluation teams where the experience of cytopathologists is limited, the widespread use of calcitonin testing demands consideration even if MTC is expected with a low prevalence rate.

## Figures and Tables

**Table 1:** Comparison of clinical data and ultrasonographic findings.

	Medullary cancer (MTC)	Other carcinomas	Benign	Difference between MTC and	
				other malignancies	Benign lesions
Number of patients	23	463	4,115		
Sex ratio (M : F)	7 : 16	59 : 404	380 : 3,735	*P* < 0.05	*P* < 0.01
	30% : 70%	13% : 87%	9% : 91%		
Median age (range) (years)	58 (20–79)	48 (16–83)	51 (12–87)		
Suspicious clinical appearance	10 (44%)	122 (26%)	170 (4.1%)	n.s.	*P* < 0.01

Ultrasonography					

Number of nodules analyzed	23	463	7695		
Median volume of nodule (mL) (range)	7.31 (0.04–67.6)	1.26* (0.01–66.8)	7.41 (0.15–172.7)		
Hypoechogenic nodule	23 (100%)	370 (80%)	4,374 (57%)	n.s.	*P* < 0.01
Microcalcifications	3 (13%)	78 (17%)	324 (4.2%)	n.s.	n.s.
Irregular borders	3 (13%)	37 (8.0%)	78 (1.0%)	n.s.	*P* < 0.01
Irregular patchy hyperechogenic areas	11 (48%)	4 (0.9%)	118 (1.5%)	*P* < 0.01	*P* < 0.01
Disturbed intranodular vascular pattern	1/14	31/268	60/5,425	*P* < 0.05	n.s.

*Anaplastic cancer not included.

**Table 2:** Cytohistological comparison in medullary and nonmedullary thyroid carcinomas.

Cytology	*N*	Histopathology		
		Medullary cancer *N* (%)	Other malignancies *N* (%)	Benign *N* (%)
Benign	3034	2 (0.07%)	31 (1.02%)	3001 (98.9%)
Suspicious	891	13 (1.46%)	164 (18.4%)	714 (80.1%)
Malignant	253	8 (3.16%)	242 (95.7%)	3 (1.19%)
Not diagnostic	423	0	26 (6.1%)	397 (93.9%)

Total	4601	23 (0.5%)	463 (10.1%)	4115 (89.4%)
